# P2X_7_ receptor antagonism ameliorates renal dysfunction in a rat model of sepsis

**DOI:** 10.14814/phy2.13622

**Published:** 2018-02-27

**Authors:** Nishkantha Arulkumaran, Marije L. Sixma, Sean Pollen, Elias Ceravola, Elisa Jentho, Maria Prendecki, Paul S. Bass, Frederick. W. K. Tam, Robert J. Unwin, Mervyn Singer

**Affiliations:** ^1^ Bloomsbury Institute of Intensive Care Medicine University College London London United Kingdom; ^2^ Division of Medicine Department of Nephrology University College London London United Kingdom; ^3^ Imperial College Renal and Transplant Centre Hammersmith Hospital London United Kingdom; ^4^ Department of cellular pathology Royal Free hospital London United Kingdom

**Keywords:** Acute kidney injury, inflammasome, sepsis

## Abstract

Sepsis is a major clinical problem associated with significant organ dysfunction and high mortality. The ATP‐sensitive P2X_7_ receptor activates the NLRP3 inflammasome and is a key component of the innate immune system. We used a fluid‐resuscitated rat model of fecal peritonitis and acute kidney injury (AKI) to investigate the contribution of this purinergic receptor to renal dysfunction in sepsis. Six and 24 h time‐points were chosen to represent early and established sepsis, respectively. A selective P2X_7_ receptor antagonist (A‐438079) dissolved in dimethyl sulfoxide (DMSO) was infused 2 h following induction of sepsis. Compared with sham‐operated animals, septic animals had significant increases in heart rate (−1(−4 to 8)% vs. 21(12–26)%; *P* = 0.003), fever (37.4(37.2–37.6)°C vs. 38.6(38.2–39.0)°C; *P* = 0.0009), and falls in serum albumin (29(27–30)g/L vs. 26(24–28); *P* = 0.0242). Serum IL‐1*β* (0(0–10)(pg/mL) vs. 1671(1445–33778)(pg/mL); *P* < 0.001) and renal IL‐1*β* (86(50–102)pg/mg protein vs. 200 (147–248)pg/mg protein; *P* = 0.0031) were significantly elevated in septic compared with sham‐operated animals at 6 h. Serum creatinine was elevated in septic animals compared with sham‐operated animals at 24 h (23(22–25) *μ*mol/L vs. 28 (25–30)*μ*mol/L; *P* = 0.0321). Renal IL‐1*β* levels were significantly lower in A‐438079‐treated animals compared with untreated animals at 6 h (70(55–128)pg/mg protein vs. 200(147–248)pg/mg protein; *P* = 0.021). At 24 h, compared with untreated animals, A‐438079‐treated animals had more rapid resolution of tachycardia (22(13–36)% vs. −1(−6 to 7)%; *P* = 0.019) and fever (39.0(38.6–39.1)°C vs. 38.2(37.6–38.7)°C; *P* < 0.024), higher serum albumin (23(21–25)g/L vs. (27(25–28)g/L); *P* = 0.006), lower arterial lactate (3.2(2.5–4.3)mmol/L vs. 1.4(0.9–1.8)mmol/L; *P* = 0.037), and lower serum creatinine concentrations (28(25–30)*μ*mol/L vs. 22(17–27)*μ*mol/L; *P* = 0.019). P2X_7_A treatment ameliorates the systemic inflammatory response and renal dysfunction in this clinically relevant model of sepsis‐related AKI.

## Introduction

Sepsis is a common and serious global health issue, accounting for 20% of all admissions to intensive care units (ICU), and is the leading cause of death in noncardiac ICUs (Angus et al. 2001). Within the intensive care unit (ICU) up to 5% of critically ill patients require renal replacement therapy (RRT) (Bagshaw et al. 2005; Uchino et al. 2005), and sepsis is implicated in half the cases of AKI (Uchino et al. 2005). The relative risk of mortality associated with AKI requiring RRT is over sixfold compared with patients without AKI (Ricci et al. 2008). Despite this, there has been a stark failure to develop any effective therapeutic agent for sepsis or sepsis‐induced AKI.

The hallmark of severe sepsis includes inflammation, a dysregulated host response, and organ dysfunction. The NLRP3 inflammasome is integral to the innate immune system and is characterized best in immune cells, including monocytes and macrophages. While the NLRP3 inflammasome is crucial for host immunity, it is unclear if excessive activation of the inflammasome in sepsis may have detrimental effects on organ function and survival. NLRP3 inhibition or genetic deletion in sepsis models has been shown to be protective in preclinical models of sepsis (Li et al. 1995) (Mariathasan et al. 2006).

The functional significance of de novo organ‐specific inflammasome expression in systemic inflammatory diseases such as sepsis is unclear. Intrinsic renal cells demonstrate components of the inflammasome in various proinflammatory diseases, while pharmacological inhibition and/or genetic deletion have shown protective effects in many preclinical models of acute kidney injury (AKI) (Turner et al. 2014). The notion that NLRP3 inhibition may be beneficial in sepsis‐induced AKI was demonstrated by caspase‐1 deficiency in a lipopolysaccharide (LPS) model of endotoxaemia (Wang et al. 2005). Similarly, P2X_7_ knockout (KO) mice subjected to cecal ligation and puncture have an attenuated inflammatory response and reduced lung injury compared with wild‐type mice (Santana et al. 2015).

In a murine model of unilateral ureteric obstruction (UUO), P2X_7_ was upregulated in cortical tubular epithelial cells, and genetic deletion protected against macrophage infiltration, collagen deposition and apoptosis (Goncalves et al. 2006). This contrasts with the predominant *glomerular* P2X_7_ expression seen in a glomerulonephritis model, which was also protected by genetic deletion or by P2X_7_ receptor antagonism (Taylor et al. 2009). The close association between the site of renal injury and the presence of P2X_7_, along with the protective effect of a P2X_7_ antagonist or genetic deletion, strongly supports a role for P2X_7_ in AKI pathogenesis. A similar relationship may hold for sepsis‐induced AKI.

We hypothesized that upregulation of the ATP‐sensitive P2X_7_ receptor contributes to renal injury in sepsis through an inflammatory response with local production of cytokines. We aimed to define the expression of this receptor within the kidney, and its relationship with cytokine production, and renal function; we sought to assess effects of P2X_7_ antagonism in a rat model of sepsis‐induced AKI. The primary objective of this study was to evaluate the effect of a selective P2X_7_ antagonist (A‐438079) in preventing renal dysfunction (as defined by an elevated serum creatinine) in an experimental model of sepsis.

## Methods

### Ethics

All animal experiments were performed under a Home Office Project License (PPL 70/7029) and local UCL Ethics Committee approval.

### Monocyte isolation and culture

We conducted ex vivo experiments to assess the biological activity of DMSO and the specific P2X_7_ receptor antagonist (A‐438079) dissolved in DMSO. Details on monocyte isolation from male Wistar rats are detailed in the supplementary data. Monocytes require priming with LPS (signal 1) prior to release of IL‐1*β* by co‐stimulation with ATP (signal 2). The duration of LPS‐priming is variable, although 4 h has been shown to be adequate (Qu et al. 2007). Stimulation by ATP is only required for 30 min after LPS priming (Mehta et al. 2001).

Previously published data demonstrates a 3 *μ*mol/L concentration of A‐438079 blocks 75% of IL‐1*β* release from cultured peritoneal macrophages stimulated with LPS and BzATP (3 *μ*g/mL LPS priming for 2 h followed by 30 min stimulation with 0.3–3.0 *μ*mol/L BzATP) (McGaraughty et al. 2007). A quantity of 10 *μ*mol/L of A‐438079 was selected for this experiment to ascertain any effect of the drug over and above that of DMSO. Experiments were repeated with at least 5 replicates per condition.

### P2X_7_ receptor antagonist pharmacokinetics

The P2X_7_ antagonist (A‐438079) was kindly donated by Abbvie (Abbott Park, IL). Based on previously published data and personal communication with Abbvie, the pharmacokinetic profile of A‐438079 was determined. A‐438079 blocked BzATP (10 *μ*mol/L)–mediated changes in intracellular Ca^++^ concentrations at rat P2X_7_ receptors but not at other P2 receptors (McGaraughty et al. 2007).

Unpublished data from Abbvie Pharmaceuticals demonstrates that the half‐life of intravenous A‐438079 is 0.69 h (41 min). An intravenous dose of 10 *μ*g/kg can achieve a peak serum concentration of 4000 ng/mL. We aimed for a peak concentration of at least 3 *μ*mol/L which would block 75% of IL‐1*β* release from macrophages in vitro. An intravenous bolus of 10 *μ*g/kg (giving a peak concentration of 4000 ng/mL) would achieve therapeutic levels in serum. We therefore administered a dose of 10 *μ*g/kg. The antagonist was given as a single bolus to achieve a peak (therapeutic) concentration, followed by 4× the dose (i.e.,: 40 *μ*g/kg) for the first half‐life, followed by 2× the dose for the second half life, followed by 10 *μ*g/(kg·h) thereafter.

### Dimethylsulfoxide

On dissolving of A‐438079 in water for a final concentration of 20 *μ*mol/L, the pH at room temperature fell to 2.2, and at 38°C was 1.6. Dimethylsulfoxide (DMSO) was therefore selected as a drug solvent. Due to its amphipathic properties, DMSO is an effective solvent for water‐insoluble compounds and is a hydrogen‐bound disrupter. As such it is a commonly used solvent for many drugs in pharmacological studies. This is a commonly used drug solvent, however, itself has biological activity (see Discussion). The strength of DMSO used varied from 3 to 15% depending on the concentration of P2X_7_ required.

### Animal model of sepsis

Male Wistar rats (Charles River, Margate, United Kingdom) weighing 300–375 g were used throughout. All experiments were performed in accordance with relevant guidelines and regulations and are detailed in the supplementary data and our characterization manuscript (Arulkumaran et al. 2017). The 72 h characterization study informed time points for the antagonist study (Arulkumaran et al. 2017). Serum creatinine was significantly elevated at 24 h in septic animals compared with sham animals. Therefore, 24 h was used as a predetermined time point.

In vivo activity was defined as the ability of A‐438079 to reduce renal and systemic IL‐1*β* release in the rat model of sepsis. Serum IL‐1*β* was elevated by 3 h and remained elevated for 48 h, whereas renal IL‐1*β* was elevated at 6 h (Arulkumaran et al. 2017). Six hours was selected as a time point to assess biological activity of A‐438079. As urinary IL‐18 was only elevated after a rise in serum creatinine, this was not used as an endpoint, since the aim was to prevent the onset of AKI in sepsis.

Drug treatment was commenced 2 h after the induction of sepsis. It was necessary to dissolve the P2X_7_ antagonist in DMSO to achieve sufficient concentration for in vivo injection. Since DMSO itself has anti‐inflammatory effects, we included DMSO injected rats as controls.

Experimental groups (*n* = 6–12 per group) initially comprised sham‐operated and untreated septic animals at both 6 h and 24 h time points, and DMSO ± A‐438079 treatment at 6 h and 24 h. On review of the results, we undertook further studies where twice the dose of A‐438079 was administered.

### Immunohistochemistry

Histological sections from the in vivo experiments were analyzed for P2X_7_ expression and evidence of acute tubular injury. Details of methods for immunohistological analyses are included in the supplementary data. For each section, ten random fields of view were analyzed and mean fluorescence intensity calculated. One section from each sample was incubated with antibody diluent instead of primary antibody and served as a control.

### ELISA

DuoSet ELISA kits (R&D Systems, Minneapolis, MN) were used according to the manufacturer's instructions. The antibody supplied in the IL‐1*β* kit does not cross‐react with pro–IL‐1*β*. ELISA was performed to assess the presence of cytokines within serum and renal tissue homogenate. Tissue cytokine levels were expressed relative to the total protein content. Absorbance was read at 450 nm using a spectrophotometric ELISA plate reader (Anthos HTII; Anthos Labtec, Salzburg, Austria).

### Western blot

Protein was extracted and estimated from whole kidney tissue. Four animals were randomly selected from each of the sham and sepsis groups, for analysis. Kidney homogenate were assessed for expression of Caspase‐1, and IL‐1*β* in sham and septic animals at 24 h. Details of methods for Western blot are included in the supplementary data.

### Statistics

The number of antagonist‐treated animals and placebo‐treated controls was calculated based on previous laboratory experience. We regarded a 20% change in a tested variable as an important biological effect. The study was designed to have a power of 90% and a significance level of 5%. At least 6 replicate animals per group per time point.

Graphpad Prism (GraphPad Software, Version 5.0d) was used for statistical analyses and graphs. All data were regarded as nonparametric due to small sample sizes (6–12 per group). Continuous variables are presented as median (interquartile range). Data were analyzed in prospectively defined groups. All 6 h experiments were analyzed as a predefined group, as were all 24 h experiments. For comparison of continuous variables between more than two groups, Kruskal–Wallis test with post hoc Dunn's test is used. Mann–Whitney *U* test was used for comparison of continuous variables between 2 groups. A *P* < 0.05 was taken as statistically significant.

## Results

### Cell culture

**Figure 1:**
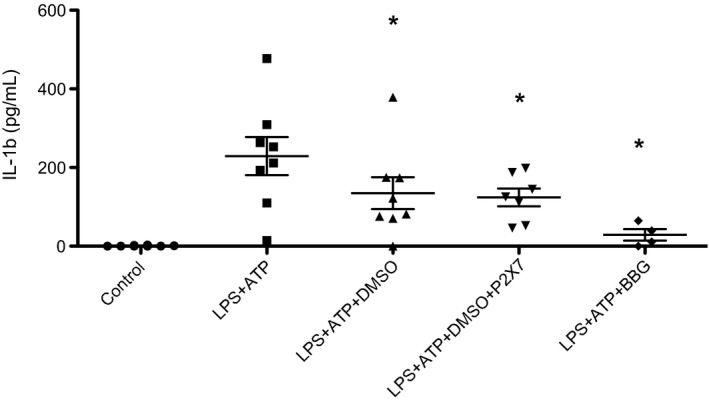
Effect of DMSO and P2X_7_ antagonist on monocyte IL‐1*β* release. LPS priming followed by ATP stimulation resulted in significant IL‐1*β* release from PBMCs. IL‐1*β* release was inhibited by the addition of 2% DMSO prior to ATP exposure. Addition of 10 *μ*mol/L P2X_7_ antagonist had no added effect on the reduction in IL‐1*β* release. 5 *μ*mol/L BBG had a significant inhibitory effect on monocyte IL‐1*β* release. Experiments were conducted twice. Data plotted represents mean and standard deviation. (*P* < 0.05 compared with LPS + ATP).

LPS and adenosine triphosphate (ATP) stimulation of cultured monocytes resulted in significant IL‐1*β* production (*P* = 0.029). Addition of 2% DMSO significantly inhibited the release of IL‐1*β* (*P* = 0.025). Addition of 10 *μ*mol/L A‐438079 had no added effect on the reduction in IL‐1*β* release. 5 *μ*mol/L brilliant blue G (BBG) had a significant inhibitory effect on monocyte IL‐1*β* release (*P* = 0.029) (Fig. 1).

### Characterization

**Figure 2:**
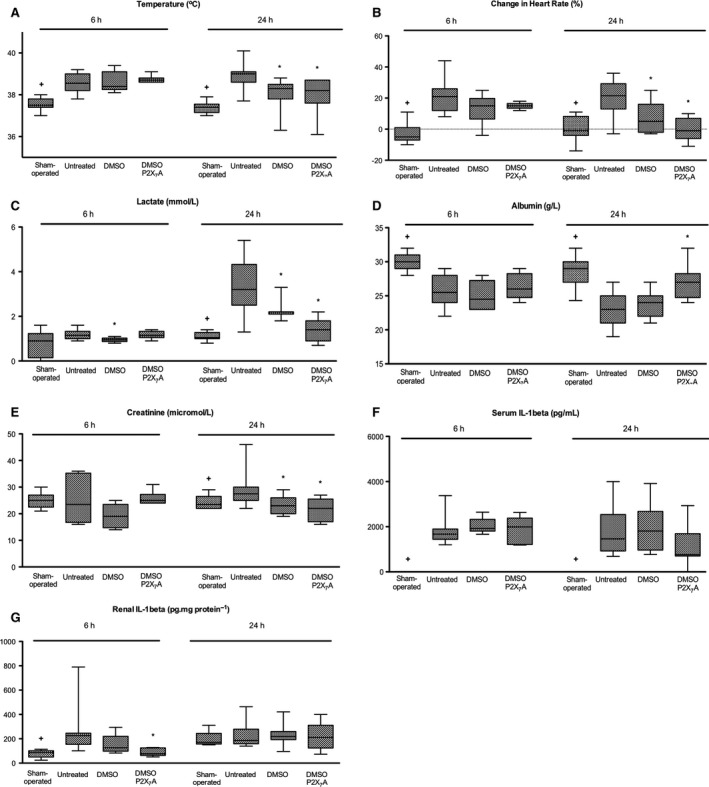
Systemic variables following induction of experimental sepsis in Wistar rats (that have received either no drug, DMSO, or A‐438079 (P2X_7_ antagonist) in DMSO) at 6 h and 24 h. Box plots represent median and interquartile range and whiskers represent minimum and maximum, respectively. Experiments include at least 6 animals per group for 6 h time point and 10–12 animals per group for 24 h time point. Septic animals have a significantly elevated body temperature and tachycardia, at 6 h and 24 h compared with sham‐operated animals. Serum albumin falls by 24 h and remains low at 24 h, whereas lactate is significantly elevated at 24 h in septic animals compared with sham‐operated animals. A‐438079 treatment reduced fever at 6 h, and improved resolution of tachycardia, serum albumin, and lactate at 24 h. Septic animals have a significantly elevated serum IL‐1*β* compared with sham animals at 6 h and 24 h. Renal IL‐1*β* Is significantly elevated by 6 h, followed by a rise in serum creatinine at 24 h. Treatment with A‐438079 after the onset of sepsis abrogated the rise in serum creatinine at 24 h with reduced renal IL‐1*β* expression at 6 h. (+*P* < 0.05 for sham vs. untreated septic animals. *P < 0.05 for treated vs. untreated septic animals).

Clinical, biochemical, and cytokine changes in the septic model are detailed in our characterization manuscript (Arulkumaran et al. 2017). Changes consistent with systemic inflammation were evident at 6 h following induction of sepsis (Table 1, Fig. 2). Compared with sham‐operated animals, septic animals had significant increases in heart rate from baseline (−1(−4 to 8)% vs. 21(12–26)%; *P* = 0.003), fever (37.4(37.2–37.6)°C vs. 38.6(38.2–39.0)°C; *P* = 0.0009), and falls in serum albumin (29(27–30)g/L vs. 26(24–28); *P* = 0.0242). Serum IL‐1*β* (0(0–10)(pg/mL) vs. 1671(1445–33778)(pg/mL); *P* < 0.001) and renal IL‐1*β* (86(50–102)pg/mg protein vs. 200 (147–248)pg/mg protein; *P* = 0.0031) were significantly elevated in septic compared with sham‐operated animals at 6 h. Serum creatinine, however, remained unchanged at 6 h (25(23–27)*μ*mol/L vs. 23(21–28) *μ*mol/L; *P* = 0.9155) in sham‐operated compared with septic animals.

**Table 1:** Physiological and biochemical variables of sham‐operated, untreated septic, and treated septic animals at 6 h and 24 h

	Groups				T‐Test	ANOVA	Post analysis Dunn's T3 test		
	Sham‐operated	Untreated Sepsis	DMSO‐ treated	DMSO/A‐438079‐ treated	Sham vs. Untreated	Untreated vs. Treated	Sepsis vs. DMSO	Sepsis vs. DMSO/A‐438079	DMSO vs. DMSO/A438079
6 h									
Change in heart rate (%)	−5 (−7−1)	21 (12–26)	15 (7–20)	15 (14–17)	0.0003	0.139	0.284	0.153	0.955
Temperature (°C)	37.4 (37.2–37.6)	38.6 (38.2–39.0)	38.4 (38.3–39.1)	38.7 (38.6–38.8)	0.0009	0.688	0.993	0.649	0.899
Albumin (g/L)	29 (27–30)	26 (24–28)	25 (23–17)	26 (25–29)	0.0242	0.62	0.790	0.961	0.589
Lactate (mmol/L)	1.1 (1.0–1.3)	1.2 (1.0–1.3)	1.0 (0.9–1.0)	1.2 (1.1–1.3)	0.4984	0.051	0.033	0.999	0.084
Creatinine (*μ*mol/L)	25 (23–27)	23 (21–28)	19 (15–25)	25 (24–27)	0.9155	0.547	0.401	0.997	0.034
Renal IL‐1*β*(pg/mg protein)	86 (50–102)	200 (147–248)	126 (98–220)	70 (55–128)	0.0031	0.077	0.372	0.021	0.372
Serum IL‐1*β*(pg/mL)	0 (0–10)	1671 (1445–3378)	1924 (1806–2325)	1989 (1214–2362)	<0.0001	0.749	0.807	1.00	0.840
24 h									
Change in heart rate (%)	−1 (−4–8)	22 (13–36)	5 (−2–16)	−1 (−6–7)	0.0015	<0.001	0.047	0.019	0.769
Temperature (°C)	37.5 (37.4–37.8)	39.0 (38.6–39.1)	38.3 (37.8–38.5)	38.2 (37.6–38.7)	0.0002	0.011	0.031	0.024	0.996
Albumin (g/L)	30 (29–31)	23 (21–25)	24 (22–25)	27 (25–28)	0.0003	0.002	0.785	0.006	0.016
Lactate (mmol/L)	0.9 (0.2–1.2)	3.2 (2.5–4.3)	2.2 (2.1–2.2)	1.4 (0.9–1.8)	0.0004	<0.001	0.037	0.001	0.001
Creatinine (*μ*mol/L)	23 (22–25)	28 (25–30)	23 (20–26)	22 (17–27)	0.0321	0.005	0.047	0.019	0.769
Renal IL‐1*β*(pg/mg protein)	168 (154–176)	186 (149–280)	219 (193–260)	224 (147–348)	0.953	0.980	0.999	1.000	0.996
Serum IL‐1*β*(pg/mL)	0 (0–10)	1463 (927–2541)	1809 (964–2678)	768 (702–1690)	<0.0001	0.141	1.000	0.261	0.207

At 24 h, septic animals had persistent changes of systemic inflammation (Table 1, Fig. 2). Compared with sham‐operated animals, septic animals had significant increases in heart rate from baseline (−5(−7 to 1)% vs. 22(13–36)%; *P* = 0.0015), fever (37.5(37.4–37.8)°C vs. 39.0(38.6–39.1)°C; *P* = 0.0002), and fall in serum albumin (30(29–31)g/L vs. 23(21–25)g/L; *P* = 0.0003). Serum IL‐1*β* (0(0–10)(pg/mL) vs. 1463(927–2541)(pg/mL); *P* < 0.001) remains significantly elevated in septic compared with sham‐operated animals at 24 h although renal IL‐1 *β* levels were similar (168(154–176)pg/mg protein vs. 186(149–280)pg/mg protein; *P* = 0.053). Serum creatinine was significantly elevated in septic animals compared with sham‐operated animals at 24 h (23(22–25)*μ*mol/L vs. 28 (25–30)*μ*mol/L; *P* = 0.0321).

**Figure 3:**
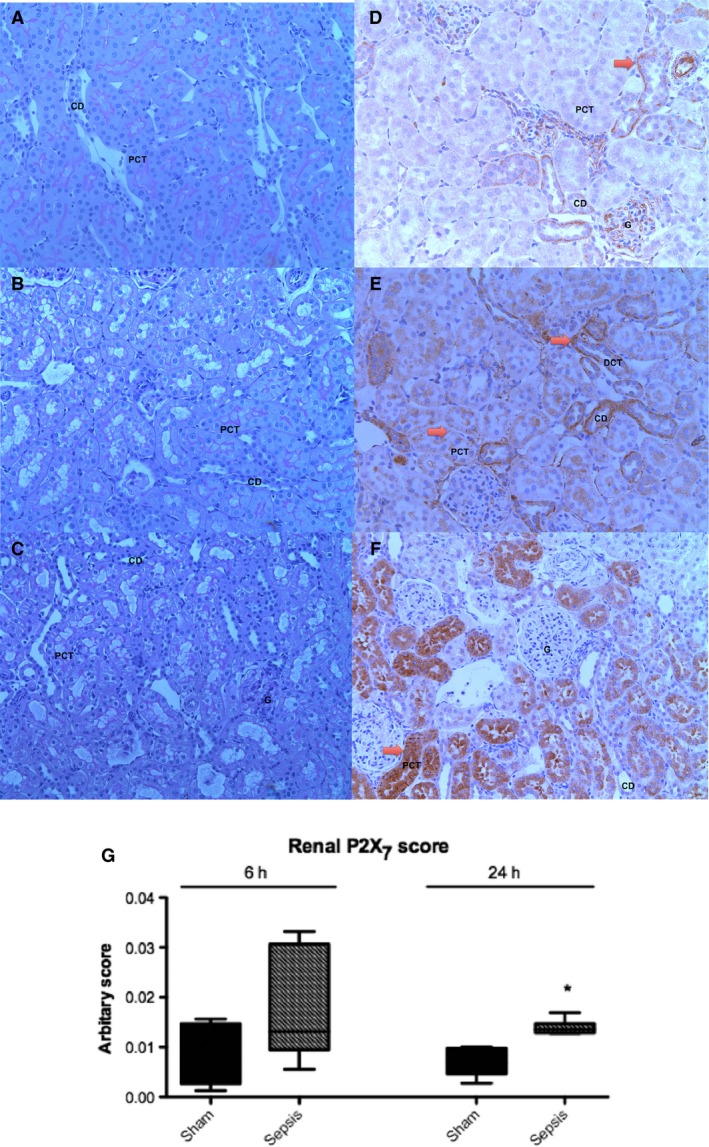
Histological analysis of kidney sections from naïve, 6 h septic, and 24 h septic animals. All images taken at x20 magnification. (A) Naive kidney with no tubular injury (B) Kidney demonstrating minimal tubular injury 6 h following induction of sepsis (C) Kidney demonstrating minimal tubular injury 24 h following induction of sepsis (D) Naive kidney with minimal tubular P2X_7_, (E) Kidney from a 6 h septic animal with minimal P2X_7_, mainly within the basolateral regions of tubules (F) Kidney from a 24 h septic animal with a similar pattern as the 6 h septic animals but greater intensity and wider distribution (G) Immunohistochemistry image analysis units for P2X_7_ expression in sham and septic animals (*n* = 6–8 per group). There is significantly increased renal tubular P2X_7_ expression at 24 h in septic animals compared with sham animals. Box plots represent median and interquartile range and whiskers represent minimum and maximum. (*P < 0.05 sham vs. septic animals). G, glomerulus; PCT, proximal convoluted tubule; DCT, distal convoluted tubule; CD, collecting duct; Arrow, P2X_7_ staining.

There was histological evidence of focal tubular injury as evidenced by flattening of tubular epithelia and tubular cell vacuolation in septic animals at 24 h (Fig. 3). Changes were minimal and sporadic. Naïve and sham‐operated animals showed minimal staining for P2X_7_ on immunohistochemistry: there was minimal constitutive P2X_7_ expression limited to distal tubular epithelial cells (Fig. 3). Increased renal P2X_7_ expression was detected in both proximal tubules and distal tubules, and was localized to the cytoplasm in septic compared with sham‐operated animals. The increased renal P2X_7_ expression reached statistical significance by 24 h (0.015 ± 0.002 vs. 0.007 ± 0.003; *P* = 0.003) (Fig. 3).

**Figure 4:**
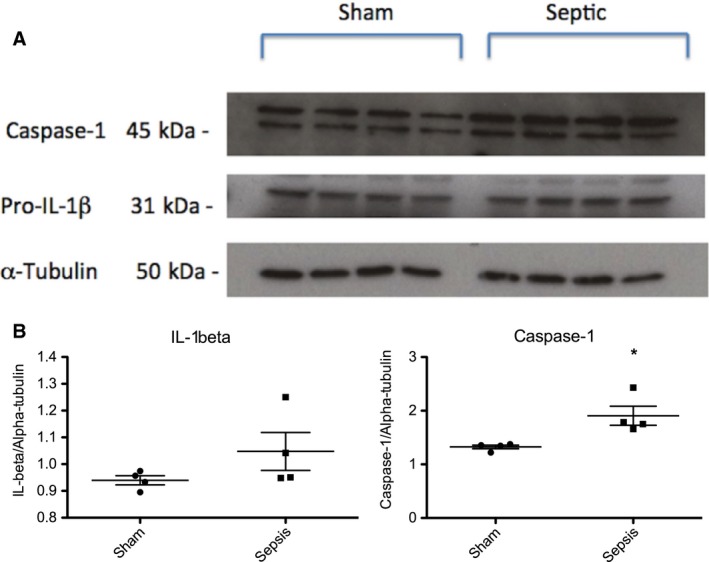
Western blot of whole kidney homogenate from sham‐operated or septic rats at 24 h. (A) Representative western blot showing the expression of renal Caspase‐1, and IL‐1*β* protein at 24 h in sham‐operated and septic animals. (B) Quantification band intensity from WB by densitometry. Septic animals have increased renal Caspase‐1 expression compared with sham operated animals (*P < 0.05 compared with sham).

Western blot of whole kidney homogenate at the 24 h time point revealed increased renal Caspase‐1 (relative expression 1.77(1.67–2.27) vs. 1.35(1.25–1.37); *P* = 0.029) protein expression in septic animals compared with sham‐operated animals (Fig. 4).

### Effect of A‐438079 (in DMSO vehicle)

Initial in vivo experiments targeting a peak concentration of 3 *μ*mol/L did not result in any clinical or biochemical differences between untreated and A‐438079‐treated septic animals (data not shown). We therefore undertook further studies where twice the dose of A‐438079 was administered.

At 6 h there was no difference among the groups in core temperature, change in heart rate, serum creatinine, or serum IL‐1*β* and between treated and untreated animals (Table 1, Fig. 2). Renal IL‐1*β* levels were significantly lower in A‐438079‐treated animals compared with untreated animals at 6 h (70(55–128)pg/mg protein vs. 200(147–248)pg/mg protein; *P* = 0.021). Arterial lactate concentration was different between DMSO‐treated and untreated animals (1.0 (0.9–1.0)mmol/L vs. 1.2(1.0–1.3)mmol/L; *P* = 0.033), although remained within the normal clinical range in all groups of animals.

At 24 h, there were a number of significant differences between A‐438079 treated animals and untreated septic animals (Table 1, Fig. 2). Compared with untreated animals, A‐438079‐treated animals had more rapid resolution of tachycardia (22(13–36)% vs. −1(−6 to 7)%; *P* = 0.019) and fever (39.0(38.6–39.1)°C vs. 38.2(37.6–38.7)°C; *P* < 0.024), higher serum albumin (23(21–25)g/L vs. (27(25–28)g/L); *P* = 0.006), lower arterial lactate (3.2(2.5–4.3)mmol/L vs. 1.4(0.9–1.8)mmol/L; *P* = 0.037), and lower serum creatinine concentrations (28(25–30)*μ*mol/L vs. 22(17–27)*μ*mol/L; *P* = 0.019).

At 24 h, compared with DMSO‐treated animals, A‐438079‐treated animals had higher serum albumin (24(22–25)g/L vs. 27(25–28)g/L; *P* = 0.016), and lower arterial lactate (2.2 (2.1–2.2)mmol/L vs. 1.4(0.9–1.8)mmol/L; *P* = 0.001). Serum IL‐1*β* and renal IL‐1*β* were similar in A‐438079‐treated, DMSO‐treated, and untreated animals at 24 h.

## Discussion

Our fluid‐resuscitated rat model of sepsis recapitulates many of the hemodynamic, biochemical, and inflammatory features seen in clinical sepsis. Various components of the NLRP3 inflammasome, including P2X_7_ and IL‐1*β*, are expressed de novo within the renal tubular epithelial cells in this septic model. This is consistent with published data showing that LPS‐primed Madin–Darby canine kidney (MDCK) renal tubular epithelial cells express Toll‐like receptor 4, NLRP3, caspase‐1, and IL‐1*β* mRNA (Jalilian et al. 2012). Tubular injury in sepsis is subtle and focal, despite significant renal dysfunction (Langenberg et al. 2008; Lerolle et al. 2010). Changes in renal function in sepsis are likely to be functional, rather than due to structural injury or hemodynamic insufficiency, with reductions in renal tubular epithelial ion channel or transporter expression in response to inflammatory stimuli, including IL‐1*β* (Schmidt et al. 2007).

Most preliminary studies using knockout mice have demonstrated a potential benefit of P2X_7_ inhibition/genetic deletion in sepsis (Santana et al. 2015; Savio et al. 2016, 2017). However, others have demonstrated increased mortality with *E. coli* urosepsis in P2X_7_‐deficient mice (Greve et al. 2017). However, the role of a selective P2X_7_ receptor inhibitor has not been evaluated in a clinically relevant model of sepsis in which treatment is administered following the onset of sepsis. We demonstrate that P2X_7_ inhibition with A‐438079 after the onset of sepsis reduced renal IL‐1*β* early expression and abrogated the rise in serum creatinine concentration at 24 h. In addition, A‐438079 treatment reduced fever and improved resolution of tachycardia, increased serum albumin and reduced lactate levels at 24 h.

### Serum IL‐1*β* with A‐438079 treatment

Other investigators have found that P2X_7_‐deficient mice produce less IL‐1*β* in response to cecal ligation and puncture (Santana et al. 2015; Savio et al. 2016, 2017). Despite treatment with A‐438079, we did not demonstrate any reduction in serum IL‐1*β* levels at either 6 h or 24 h. A significant amount of IL‐1*β* is likely to have been released prior to the initiation of treatment with A‐438079. Moreover, IL‐1*β* release following a P2X_7_ receptor antagonist given after the induction of sepsis cannot directly be compared with similar experiments in animals with genetic deletion. Furthermore, it is unlikely that IL1‐*β* is released by only one mechanism in polymicrobial sepsis. Monocytes are capable of IL‐1*β* release independent of P2X_7_ receptor activation (e.g., via TLR 7/8 ligands), though P2X_7_ activation accelerates mature IL‐1*β* release from monocytes (Ward et al. 2010). Splenic dendritic cells are able to release mature IL‐1*β* in the absence of P2X_7_ activation, but require the NLRP3 inflammasome. Intraperitoneal administration of LPS‐induced IL‐1*β* production in serum, which was abrogated in Nlrp3‐null mice, but unaffected in P2X_7_‐deficient mice (He et al. 2013). IL‐1*β* may be released independently of NLRP3 activity. NLRP3‐deficient mice had detectable (but significantly reduced) levels of IL‐1*β* (Mariathasan et al. 2006). In fact, P2X_7_ deficient mice inoculated with *α*‐hemolysin (HlyA)‐producing Escherichia coli had markedly lower survival, higher cytokine levels (including IL‐1*β*), and activated intravascular coagulation compared with wildtype mice (Greve et al. 2017). These paradoxical findings were explained by caspase‐8‐dependent IL‐1*β* production during sepsis with uropathogenic *E. coli*.

### Inflammasome‐independent actions of P2X_7_

Despite similar serum IL‐1*β* levels at 6 h and 24 h in A‐438079‐treated and untreated animals, there were clinical features suggestive of an attenuated inflammatory response in A‐438079‐treated animals. The improvement in systemic inflammation and organ function must also be explained by mechanisms independent of the inflammasome and IL‐1*β* release. Arterial lactate at 24 h was lower with DMSO/A‐438079 treatment compared with untreated animals, suggesting improved mitochondrial function. Prevention of excessive P2X_7_ activation may have promoted mitochondrial health associated with improved oxidative phosphorylation and lower lactate. Basal expression of P2X_7_ promotes cell growth in P2X_7_‐transfected HEK293 (Adinolfi et al. 2005). However, an ATP challenge resulted in mitochondrial fragmentation mediated by increases in ER Ca^2+^ concentration and subsequent cell death (Adinolfi et al. 2009).

The reduction in serum albumin levels in sepsis is due in part to increased microvascular permeability/capillary leak. Extracellular ATP and ADP activate NF‐*κ*B and induce endothelial cell apoptosis mediated via the P2X_7_R (von Albertini et al. 1998). Animals treated with A‐438079 had a significantly higher serum albumin compared with untreated animals, which may have been mediated by improved endothelial barrier function.

### Systemic versus local IL‐1*β* production

There were systemic effects of A‐438079 treatment in sepsis over and above renal‐specific effects. At 24 h, animals treated with A‐438079 had a lower temperature and more rapid resolution of tachycardia compared with untreated animals. The resolution of tachycardia at 24 h associated with A‐438079 might be explained by a reduction in cardiac inflammatory mediator levels.

It is difficult to ascertain the relative contribution of systemic versus renal inflammasome activation in sepsis‐induced kidney dysfunction, with variable evidence in the literature. Mice with functional TLR4 deficiency (C3H/HeJ mice) transplanted with wild‐type kidneys were protected from LPS‐induced AKI, whereas wild‐type mice transplanted with C3H/HeJ kidneys developed severe LPS‐induced AKI. This suggests that TLR4 expression in circulating cells propagates injury in septic AKI, rather than intra‐renal TLR4 (Cunningham et al. 2004). In contrast, chimeria mice deficient of renal TLR4 but with wild‐type bone marrow were protected against LPS‐induced renal proximal tubular oxidative stress. Chimera mice generated through bone marrow transfer from TLR4 KO into WT recipient (KO/WT), however, exhibited oxidative stress similar to WT mice, suggesting a pathogenic role for renal TLR4 over systemic TLR4 (Kalakeche et al. 2011). In a murine model of UUO, use of bone marrow chimeras revealed that NLRP3 mediates injurious/inflammatory processes in both hematopoietic and nonhematopoietic cellular compartments (Vilaysane et al. 2010).

### Limitations

Various drug solvents such as ethanol and MF59 can affect the inflammasome directly (Nurmi et al. 2013) or indirectly (Seubert et al. 2011). DMSO was the only suitable vehicle for A‐438079, but it also possesses some biological activity. DMSO exerts anti‐inflammatory effects as a hydroxyl radical scavenger (Man et al. 2014). DMSO also decreases IL‐1*β* mRNA transcription and reduces inflammation (Ahn et al. 2014a). Furthermore, mitochondrial reactive oxygen species can induce IL‐1*β* and NLRP3 activity, and this is inhibited by DMSO (Ahn et al. 2014b). However, there are also isolated reports of the proinflammatory action of DMSO in human peripheral blood mononuclear cells (PBMCs) (Xing and Remick 2005) and low‐dose toxicity in a retinal neuronal cell line (Galvao et al. 2014). Differences may be related to the dose of DMSO, cell/species type, and duration of stimulation. DMSO‐treated septic animals were compared with A‐438079 in DMSO. A number of clinical and biochemical parameters were improved at 24 h with A‐438079 with DMSO compared with untreated animals. However, there are trends in improvement in temperature, resolution of tachycardia, and serum creatinine with DMSO alone, suggesting some synergy.

The changes found in animals treated with the P2X_7_ antagonist may have been due to reduced systemic inflammatory response (evidenced by less tachycardia and fever), with better perfusion to organs including the kidney, rather than specific actions over the renal‐inflammasome activation.

ELISAs were performed on whole kidney homogenate for IL‐*β* measurement, which does not allow localization to specific cell types. Immunohistochemistry would enable localization of these cytokines. Although insights into renal expression of the inflammasome and P2X_7_ in sepsis have been demonstrated, the expression of the P2X_7_ in other organs and on immune cells also needs to be evaluated.

### Summary

We demonstrate a number of strengths of our experimental model relevant to potential therapeutics in sepsis. Sepsis is associated with an increase in renal IL‐1*β* and tubular cell P2X_7_ expression by 6 h and 24 h, respectively. Treatment with A‐438079 after the onset of sepsis abrogated the rise in serum creatinine at 24 h with reduced renal IL‐1*β* expression early. In addition, A‐438079 treatment reduced fever, improved resolution of tachycardia, increased serum albumin concentration and reduced lactate at 24 h. Given these encouraging results in an in vivo model of sepsis that is relevant to human disease, further studies targeting P2X_7_ in sepsis are warranted.

## Ethics approval and consent to participate

All animal experiments were performed under a Home Office Project License (PPL 70/7029) and local University College London Ethics Committee approval.

## Conflict of Interest

Materials: Abbvie pharmaceuticals (Chicago, USA) have provided the selective P2X_7_ antagonist (A‐438079). FWKT has received research project grants from AstraZeneca Limited, Baxter Biosciences, Boehringer Ingelheim, and MedImmune. He has consultancy agreements with Rigel Pharmaceuticals, Novartis and Baxter Biosciences.

## Data Accessibility

## Supporting information




**Data S1**. Supplementary data.
