# Supplementary material for: P2X7 receptor antagonism ameliorates renal dysfunction in a rat model of sepsis
Source: Physiol Rep. 2018 Feb 27;6(5):e13622. doi: 10.14814/phy2.13622 (PMC5828936; doi:10.14814/phy2.13622)
Supplement: Supplementary file 1 — Data S1. Supplementary data. [file PHY2-6-e13622-s001.doc]

# Supplementary data

## Methods

## Monocyte isolation and culture

We conducted ex-vivo experiments to assess the biological activity of DMSO and the specific P2X_7_ receptor antagonist (A-438079) dissolved in DMSO.

Naïve male Wistar rats were given 1000 units heparin intraperitoneally 30min prior to culling. Animals were anaesthetized and blood obtained via cardiac puncture. Blood was stored in a sterile Falcon tube on ice. Fifteen ml blood was added to 30ml volume of sterile PBS. Twenty ml was transferred onto 15ml of Ficoll ensuring the Ficoll-blood layer remained intact. The suspension was centrifuged for 30min at 1400 rpm at room temperature. Three layers were formed - the lowermost Ficoll layer, followed by the thin layer of monocytes, and the layer of PBS. Cells were pipetted and re-suspended in 30 ml complete culture medium (RPMI with 10% fetal bovine serum (FBS) and 1% Penicillin/Streptomicin) and centrifuged at 1200 rpm for 8 min at room temperature. The monocyte pellet was re-suspended once again and placed in 30 ml complete medium at 37^o^C in 5% CO_2_ / 95% O_2_ for 24 h. The next day, cells were centrifuged at 1200 rpm for 8 min at room temperature, re-suspended in 2 ml serum-free culture medium, and counted. Serum-free medium was added to give a total cell count of 4 x 10^6^ cells per 1 ml. One ml was added per well in a 6 well plate and left overnight at 37^o^C in 5% CO_2_/ 95% O_2_.

The next day, cell culture was started. Monocytes require priming with LPS (signal 1) prior to release of IL-1β by co-stimulation with ATP (signal 2).

The following conditions were used:

- Control
- LPS + ATP: 1 µg/ml LPS per well incubated for 5.5 h followed by 5 nM ATP for 30 min
- LPS + ATP + DMSO/P2X_7_A: 1µg/ml LPS per well incubated for 5.5 h followed by 10 µM A-438079 dissolved in DMSO (final concentration 2%) followed by 5 nM ATP for 30 min
- LPS + ATP + DMSO: 1 µg/ml LPS per well was incubated for 5.5 h followed by DMSO (final concentration 2%) followed by 5 nM ATP for 30 min
- Positive control of brilliant blue G (BBG): 1 µg/ml LPS per well incubated for 5.5 h followed by 5 µM BBG in water and then 5 nM ATP for 30min

Previously published data demonstrates a 3 μM concentration of A-438079 blocks 75% of IL-1β release from cultured peritoneal macrophages stimulated with LPS and BzATP (3 μg/ml LPS priming for 2 h followed by 30 min stimulation with 0.3 – 3.0μM BzATP) . A relatively high dose of A-438079 was selected for this experiment to ascertain any effect of the drug over and above that of DMSO. Cell suspension was collected in a 1.5 ml tube and centrifuged at 1200g for 5 min at room temperature. Medium was transferred to new tubes and stored at -80^o^C. Experiments were repeated with at least 5 replicates per condition.

## Animal model of sepsis

The rat model was chosen because of the appropriate size of physiological monitoring. Male Wistar rats (Charles River, Margate, United Kingdom) weighing 300-375g were used throughout. All experiments were performed in accordance with relevant guidelines and regulations.

Before experimentation rats were housed in cages of six on a 12-12 h light–dark cycle. All invasive and imaging techniques were performed under general anesthesia as described previously . Following internal jugular central venous catheter (CVC) placement, rats were placed in individual cages mounted on the tether/swivel system to secure the intravenous catheter and allow unimpeded movement with free access to food and water. 24h post-CVC insertion, sepsis was induced by intraperitoneal injection of fecal slurry. A similar procedure was avoided in sham animals to prevent inadvertent bowel perforation.

The laboratory has previously determined the optimal volume and rate of fluid administration to maintain intravascular volume based on echocardiogram parameters . Transthoracic echocardiography was performed prior to CVC insertion, and at 6 and 24h as previously described. At the terminal experiment, surgical tracheostomy performed and arterial line inserted in anaesthetized, spontaneously breathing animals. Arterial blood gas analysis was performed with 0.2 ml taken into heparinized capillary tubes (ABL-70 analyser, Radiometer, Copenhagen, Denmark).

The left kidney was isolated and the upper pole was placed into formalin and the rest snap-frozen in liquid nitrogen. Cardiac puncture performed to obtain blood. The blood was placed in a heparinized tube and centrifuged at 4000g for 10 min. The serum was siphoned off, snap-frozen in liquid nitrogen, and stored at -80^o^C.

## Immunohistochemistry

Sections from the *in vivo* experiments were fixed in 10% formalin and embedded in paraffin. Sections were cut 5μm thick and then mounted on glass slides (performed by histopathology lab at Imperial College, London). For detection of P2X_7_, slides were dewaxed and rehydrated through graded xylene and ethanol, respectively. Antigen retrieval was performed by placing slides in 0.01M sodium citrate buffer heated to 90^o^C in a water bath for 15 minutes. Slides were then immersed in 50% methanol containing 0.3% H_2_O_2_ for 1h to block endogenous peroxidase activity. Slides were washed with PBS then incubated with 20% normal goat serum for 30 minutes to prevent non-specific binding. The primary antibody (mAb P2X_7_, APR-004, Alamone, Jerusalem, Israel) was diluted in 1% BSA/0.05M Tris-HCL (pH 7.2) and incubated at room temperature for 1 hour. Slides were rinsed in PBS for 15 minutes and incubated with peroxidase-labelled polymer conjugated to goat anti-mouse immunoglobulins (Dako, Ely, Cambs) for 1h. Antibody binding was visualized using 3,3’-diaminobenzidene (Dako) and counterstained with hematoxylin. Spleen tissue from a septic animal at 6 hours was used as a positive control.

Sections were examined using a light microscope (Olympus Optical, London, UK) at x20 magnification. All sections were scored in a blinded manner. Ten random fields of view of the cortex were analyzed for each section at x20 magnification. P2X_7_ staining was assessed semi-quantitatively using a Color Coolview camera (Photonic Sciences, Robertsbridge, UK) and analyzed using Image Pro Plus software (Media Cybernetics, Silver Spring, MD, USA).

## Western Blot

Protein was extracted and estimated from whole kidney tissue. Four animals were randomly selected from each of the sham and sepsis groups, for analysis. Samples (20 μg protein) were electrophoresed at 100 V for 1 h through a 12% or 15% SDS-PAGE gel under reducing conditions. Proteins were transferred to a polyvinylidine difluoride (PVDF) membrane (GE Healthcare, Amersham, Bucks) at 10V for 45 min, and then blocked for 1 h in 5% milk/1% TBST. Nonspecific binding sites were blocked in 5% non-fat milk and 0.1% Tween-20 in TBS for 1 h at room temperature.

The antibody binding was revealed using an goat anti-mouse (caspase-1) followed by HRP anti-goat IgG (Sigma) at 1:3000. In an analogous manner, membranes were probed for P2X_7_ using goat anti-mouse P2X_7_ (Santa Cruz Biotechnology, Santa Cruz, CA) at 1:1000 followed by HRP anti-goat IgG (Sigma) at 1:3000. For IL-1β, membranes were probed using goat anti-mouse IL-1β (Santa Cruz Biotechnology, Santa Cruz, CA) at 1:1000 followed by HRP anti-goat IgG (Sigma) at 1:3000. Beta actin was probed with rabbit polyclonal antibody (Abcam) at 1:1000 followed by HRP anti-rabbit IgG (Sigma) at 1:3000. Membranes were incubated in primary antibody buffer overnight shaking gently at 4 °C, followed by being washed three times in 0.1% TBST after which they were incubated with their respective peroxidase-labeled secondary antibodies at room temperature for 1 h and again washed three times.

The blots were then detected using the enhanced chemiluminescence (ECL) detection system (Amersham Life Science). Developed films were analyzed semi-quantitatively by scanning volume density using a Bio-Rad GS-690 densitometer and Image J software (NIH, USA). Optical volume density value for IL-1β, caspase-1, or P2X_7_ was corrected for loading using β-actin expression. Results were expressed as the percentage of the average control volume density. The size of target protein was determined by comparison with protein molecular weight markers (Bio- Rad Laboratories Ltd.) using the same analysis package
